# White Matter Microstructure Alterations: A Study of Alcoholics with and without Post-Traumatic Stress Disorder

**DOI:** 10.1371/journal.pone.0080952

**Published:** 2013-11-18

**Authors:** Caitlin A. Durkee, Joelle E. Sarlls, Daniel W. Hommer, Reza Momenan

**Affiliations:** 1 Section on Brain Electrophysiology and Imaging, Laboratory of Clinical and Translational Studies, National Institute on Alcohol Abuse and Alcoholism, National Institutes of Health, Bethesda, Maryland, United States of America; 2 National Institute of Neurological Disorders and Stroke, NIH MRI Research Facility, National Institutes of Health, Bethesda, Maryland, United States of America; Beijing Normal University, China

## Abstract

Many brain imaging studies have demonstrated reductions in gray and white matter volumes in alcoholism, with fewer investigators using diffusion tensor imaging (DTI) to examine the integrity of white matter pathways. Among various medical conditions, alcoholism and post-traumatic stress disorder (PTSD) are two comorbid diseases that have similar degenerative effects on the white matter integrity. Therefore, understanding and differentiating these effects would be very important in characterizing alcoholism and PTSD. Alcoholics are known to have neurocognitive deficits in decision-making, particularly in decisions related to emotionally-motivated behavior, while individuals with PTSD have deficits in emotional regulation and enhanced fear response. It is widely believed that these types of abnormalities in both alcoholism and PTSD are related to fronto-limbic dysfunction. In addition, previous studies have shown cortico-limbic fiber degradation through fiber tracking in alcoholism. DTI was used to measure white matter fractional anisotropy (FA), which provides information about tissue microstructure, possibly indicating white matter integrity. We quantitatively investigated the microstructure of white matter through whole brain DTI analysis in healthy volunteers (HV) and alcohol dependent subjects without PTSD (ALC) and with PTSD (ALC+PTSD). These data show significant differences in FA between alcoholics and non-alcoholic HVs, with no significant differences in FA between ALC and ALC+PTSD in any white matter structure. We performed a post-hoc region of interest analysis that allowed us to incorporate multiple covariates into the analysis and found similar results. HV had higher FA in several areas implicated in the reward circuit, emotion, and executive functioning, suggesting that there may be microstructural abnormalities in white matter pathways that contribute to neurocognitive and executive functioning deficits observed in alcoholics. Furthermore, our data do not reveal any differences between ALC and ALC+PTSD, suggesting that the effect of alcohol on white matter microstructure may be more significant than any effect caused by PTSD.

## Introduction

Chronic alcohol use is characterized by deficits in a multitude of cognitive and behavioral functioning, including decision-making, executive functioning, and impulsivity [1–3]. Previous brain imaging studies have shown that accompanying these deficits are abnormalities in both gray and white matter structures. Consistent findings are reduced volumes of cortical gray matter structures, such as the frontal, parietal, and temporal lobes; subcortical gray matter structures, such as the thalamus and amygdala [4–7]; and white matter structures such as the corpus callosum [7–11]. In addition to structural abnormalities, alcohol dependent subjects also present with abnormal activation patterns in functional MRI studies [12–14].

To better understand these aberrant structural and functional characteristics, the microstructural properties of implicated white matter structures should also be investigated. DTI allows one to quantitatively assess the microstructural properties of the white matter pathways by measuring the displacement of water molecules. One of the output metrics from DTI is FA, which ranges from 0, being fully isotropic, to 1, being fully anisotropic. FA reflects a complex mixture of tissue properties including coherence of fiber orientation, myelination, and axonal density [15]. Higher FA values may indicate greater coherence of fiber orientation, increased myelination, decreased axon diameter, increased axon density, and amount of intracellular and extracellular fluid [16–18]. Although it is inappropriate to interpret FA as a direct measure of white matter integrity, it does reflect the physical microstructural properties and serves as a starting point for exploring structural connectivity [15]. 

Using FA and other diffusivity measures, recent studies have shown that changes in properties of white matter pathways connecting implicated cortical and subcortical regions may also contribute to the cognitive deficits seen in alcoholics. In Harris et al. [19], lower FA in alcoholics in the right superior longitudinal fascicles II and III, orbitofrontal cortex white matter, and cingulum bundle was associated with lower working memory scores. Pfefferbaum et al. [20] showed that the greatest white matter abnormalities in alcoholics compared to healthy controls were observed in the frontal forceps, internal and external capsules, fornix, and longitudinal fasciculus, and that these were correlated with poorer performance on psychomotor speed tests. Other white matter regions typically implicated in alcoholism are the frontal and limbic pathways, centrum semiovale, and corpus callosum [11,20–24], with the genu of the corpus callosum and other more anterior white matter structures often found to be more compromised than posterior structures [20,25]. Studies have reported inconsistent findings regarding the effect of sex on white matter deficits in alcohol misuse [11,20,26].

Alcohol use disorders (AUD) are highly concomitant with other psychopathologies including PTSD. A number of imaging studies have shown reduced cingulate cortex and orbitofrontal cortex volumes [27], with inconsistent findings regarding hippocampal and amygdalar volume in individuals with PTSD [28–31]. Relatively consistent findings in DTI studies of PTSD is abnormal FA in the cingulum bundle, internal capsule, and in the white matter of the precentral gyrus [32], with most studies showing decreased FA in these areas [33–36]. Since these frontal and limbic regions are also implicated in AUD, it would be useful to elucidate any microstructural differences, if any, that exist between the two disorders. In particular, we hypothesized that alcohol dependent subjects with PTSD may present with greater FA abnormalities in the cingulum bundle, one of the structures most implicated in PTSD. This is the first study to our knowledge that examines the effects of PTSD on white matter microstructure in alcohol dependent subjects with healthy volunteers as control subjects.

This study examines whole brain white matter microstructure in three subject populations – alcohol dependent individuals (ALC), alcohol dependent individuals with PTSD (ALC+PTSD), and healthy volunteers (HV). The aims of our study were to corroborate previous DTI findings of abnormal white matter FA in fronto-limbic circuitry, in addition to looking more closely at potential differences between alcohol dependent subjects with and without PTSD. In light of previous findings, we hypothesized that both alcohol dependent groups would have lower FA values when compared to healthy volunteers. Given the overlap in neurocircuitry between alcohol dependence and PTSD, we also hypothesized that PTSD might have an additive effect by further compromising white matter FA. 

## Materials and Methods

### Ethics Statement

All recruitment, informed written consent, and testing procedures were approved by the Institutional Review Board of the National Institute on Alcohol Abuse and Alcoholism (NIAAA).

### Subjects

Each subject was recruited using public advertisements and underwent screening to check for eligibility criteria. ALC and ALC+PTSD subjects were recruited from the National Institute on Alcohol Abuse and Alcoholism (NIAAA) inpatient unit at the National Institutes of Health Clinical Center in Bethesda, MD, and were abstinent from alcohol for 15.6±6.0 and 23.8±13.2 days, respectively, at the time of their scans (p = 0.03; Table 1). All subjects were right-handed, had no other current major medical conditions, and had no remarkable brain morphology as assessed by a radiologist. HV subjects had no evidence of medical problems in medical history interviews, physician examination, or clinical chemistry panel. Axis I disorders were classified using self-report with the Structured Clinical Interview for DSM-IV [37], which was used to categorize subjects into healthy volunteers (HV), alcohol dependent subjects (ALC), and alcohol dependent subjects with PTSD (ALC+PTSD). HV subjects had no current Axis I disorders. ALC subjects were required to meet criteria for current alcohol dependence, while criteria for other Axis I disorders could also have been met. ALC+PTSD subjects were additionally required to meet criteria for current PTSD (Table S1). Cognitive testing was performed using The Wechsler Adult Intelligence Scale - Revised (WAIS-R) [38] and The Wechsler Abbreviated Scale of Intelligence (WASI) [39]. All subjects produced negative urine drug screens and negative breath-alcohol readings at the time of the study.

**Table 1:** Demographic information on healthy volunteers (HV), alcohol dependent subjects without PTSD (ALC), and alcohol dependent subjects with PTSD (ALC+PTSD).

**Demographics of study populations**	**HV (n=19)**	**ALC (n=19)**	**ALC+PTSD (n=17)**	**Statistics**	**Statistics between ALC and ALC+PTSD**
**Mean Age (SD^a^)**	26.22 (4.05)	32.61 (7.30)	37.17 (8.69)	^b^ P < 0.0001	^b^ P = 0.10
**Number of Females**	9	5	8	^c^ P = 0.31	^c^ P = 0.19
**Years of Education (SD)**	16.47 (1.98)	12.68 (2.79)	13.71 (1.65)	^b^ P < 0.0001	^b^ P = 0.20
**Mean of Average Drinks Past 30 Days (SD)**	17.46 (16.12)	382.68 (281.70)	250.58 (178.36)	^b^ P < 0.0001	^b^ P = 0.11
**Mean No. Days Abstinent before Scan Date (SD)**	--	15.56 (6.00)	23.79 (13.17)	--	^b^ P = 0.025
**Mean Early Life Stress Questionnaire Score (SD)**	0.37 (0.60)	2.89 (2.11)	5.59 (3.50)	^b^ P < 0.0001	^b^ P = 0.0077
**Mean CAPS Score, week prior**	--	--	2.77 (0.83)	--	--
**Number of Smokers**	0	10	11	^c^ P < 0.0001	^c^ P = 0.46
**Current Cocaine Abuse and/or Dependence**	--	1	2	--	--
**Current Cannabis Abuse and/or Dependence**	--	1	1	--	--
**Current Major Depressive Disorder**	--	2	3		

^a^ SD = standard deviation; ^b^ ANOVA; ^c^ Likelihood Ratio Chi^2^ test

ALC subjects (n=19; age 21-44, mean 32.61±7.3; 14 males) met current DSM-IV criteria for alcohol dependence, while ALC+PTSD subjects (n=17; age 21-48, mean 37.17±8.69; 9 males) met current DSM-IV criteria for both alcohol dependence and PTSD. HV subjects (n=19; age 22-37, mean 26.2±4.1; 10 males) did not meet criteria for any current DSM-IV diagnosis. HV subjects were significantly younger than All_ALC subjects (p < 0.0001), but mean age between ALC and ALC+PTSD was not significantly different (p = 0.10; Table 1).

### DTI acquisition

Subjects were scanned with a 3 Tesla MRI scanner (General Electric, Milwaukee, WI) using a 16-channel head coil. Whole brain DTI scans were acquired using a single-shot EPI sequence with the following parameters: TR = 13000 ms, TE = set to minimum (range: 83-85 ms), slice thickness = 2.4 mm, 54 slices, field of view = 24 cm, acquisition matrix = 96x96, reconstruction matrix = 256x256, maximum B value = 1000 s/mm^2^, with 33 diffusion directions and 3 non-diffusion-weighted images (b-value = 0 image). A high-resolution whole brain T2 image, with the same slice thickness, was used to register the tensor data to structural volumes.

### DTI data processing

DTI data was processed using TORTOISE [40]. The pre-processing in TORTOISE included rigid body motion correction as well as eddy-current distortion correction. The diffusion tensor was calculated using non-linear fitting, along with computed tensor-derived quantities, including FA.

### Tract-Based Spatial Statistics analysis

The FA data computed from TORTOISE was analyzed using Tract-Based Spatial Statistics (TBSS v1.2) [41], a statistical analysis method in FMRIBS Software Library (FSL) [42]. All subject FA data in each comparison were aligned and warped to MNI152 standard space using a nonlinear registration. The mean FA image was then used to generate a mean FA skeleton, which delineated the centers of all white matter tracts common to all subjects within the analysis. The voxel-wise cross-subject statistical analysis (familywise error corrected for multiple comparisons across space and threshold set at p < 0.05) was performed using the results of each subject’s FA data being projected onto this mean FA skeleton. TBSS performed a voxel-wise statistical analysis for the following comparisons: HV versus ALC, HV versus ALC+PTSD, ALC versus ALC+PTSD, HV versus ALC and ALC+PTSD combined (hereafter All_ALC), as well as the reverse comparison of each (e.g. ALC versus HV). 

### ROI analysis

A post-hoc ROI analysis was performed on structures that showed group FA difference in the TBSS analysis to provide us a greater understanding of the TBSS results. Specifically, the ROI analysis revealed whether the group FA differences remained after correcting for multiple covariates. We examined FA in two separate JMP-SAS (SAS Institute Inc., Cary, North Carolina) analyses: 1) a “two-group” analysis, comparing HV and All_ALC, and 2) a “three-group” analysis, comparing HV versus ALC versus ALC+PTSD. 

The seven ROIs included the body of the fornix, anterior commissure, genu of the corpus callosum, splenium of the corpus callosum, the right inferior fronto-occipital fasciculus (IFO), and the bilateral dorsal cingulum bundle. WM structures were determined by the JHU White-Matter Tractography Atlas and confirmed by an experienced investigator. Each of these areas, aside from the anterior commissure, has previously been implicated in DTI studies of alcoholism and PTSD. Only the right IFO was selected in the ROI analysis since significance in the left IFO did not survive the p < 0.05 threshold and multiple comparison-corrected TBSS analysis. Using MIPAV (NIH, Bethesda, MD), each ROI was created from a particular region of the mean FA skeleton generated from TBSS. The mean FA of each ROI for each individual subject was then computed. Using JMP-SAS, an analysis of variance (ANOVA) was performed on both the two-group and three-group analyses correcting for age, sex, years of education, and a group by sex interaction, as these factors have been found to influence white matter FA. We used years of education rather WAIS-R and WASI scores since three subjects’ scores were not available. Years of education was later removed as a covariate in the GLM analysis, however, since it was never a main effect in any ROI analysis. To avoid the effect of outliers, we excluded any ROI’s mean voxel FA values that were greater than two standard deviations away from the average mean-FA within each group (total of 17 out of 385 from both the two-group and three-group analyses). Post-hoc Student’s *t* tests were used to examine FA differences amongst groups. 

## Results

### TBSS Results

**Figure 1:**
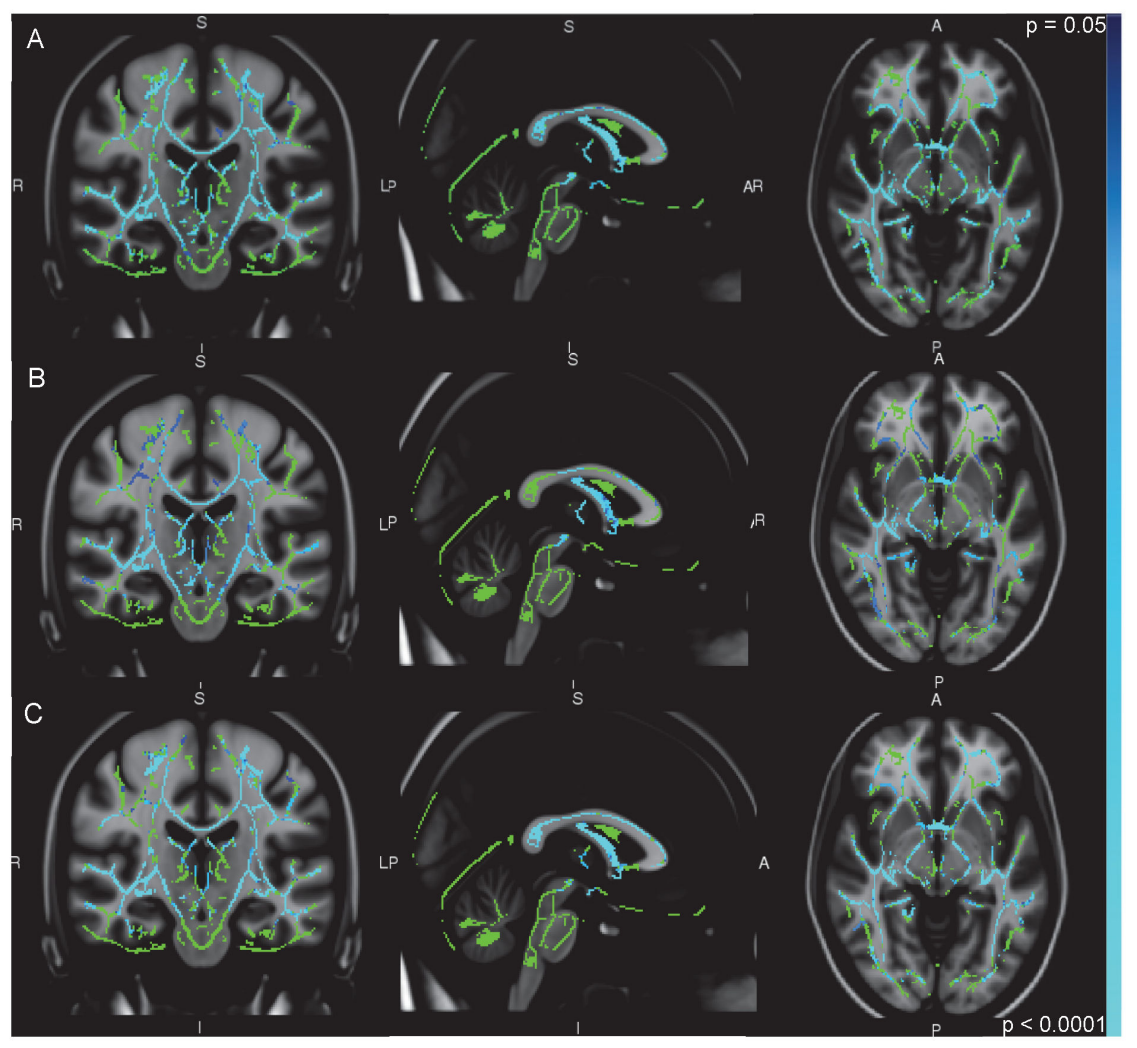
TBSS results from A) HV>All_ALC B) HV>ALC, and C) HV>ALC+PTSD comparisons. Blue and light blue indicate significant differences (threshholding at p < 0.05) superimposed on a green FA skeleton and overlaid on an MNI template.

In the HV > All_ALC (Figure 1A), HV > ALC (Figure 1B), and HV > ALC+PTSD (Figure 1C) comparisons, FA in HV was significantly higher in white matter structures in the frontal, limbic, temporal, and parietal lobes, including the fornix, anterior commissure, corpus callosum, cingulum bundle, and the left IFO. The HV > ALC+PTSD and HV > All_ALC contrasts visibly revealed more areas of greater significant difference than the HV > ALC contrast. The ALC > ALC+PTSD and ALC+PTSD > ALC contrasts, however, showed no areas of significant difference. In no area was FA higher in the ALC, ALC+PTSD, or All_ALC groups when compared with HV. 

### Region of Interest (ROI) Results

All statistics reported in the parentheses below are the result of an Effects Test on all of the covariates used in the GLM (i.e. group, sex, age, and a group by sex). Post-hoc Student’s *t*-tests show what groups, if any, were different. Tables 2 and 3 also display ROI results of the two-group and three-group analyses, respectively. Note that the tables report only the effect of group on FA, in addition to the whole model GLM result.

**Table 2:** Statistics of two-group Region of Interest analysis: HV vs All_ALC.

**Region of Interest**	**Whole Model** ^a^ **F ratio, Prob > F**	**Student’s t**			**% Difference in FA**
		**Group**	**Level** ^b^	**Least Square Mean** (**SE** ^c^) **of FA**	**HV > All_ALC**
**Anterior Commissure**	4.21, 0.0053	HV	A	0.39 (0.020)	20.5
		All_ALC	B	0.31 (0.014)	
**Fornix**	6.33, 0.0004	HV	A	0.74 (0.019)	14.9
		All_ALC	B	0.63 (0.013)	
**Genu**	4.02, 0.0070	HV	A	0.84 (0.012)	3.6
		All_ALC	A	0.81 (0.008)	
**Splenium^+^**	5.57, 0.0009	HV	A	0.86 (0.013)	1.2
		All_ALC	A	0.85 (0.010)	
**Right IFO**	12.49, <0.0001	HV	A	0.62 (0.011)	9.7
		All_ALC	B	0.56 (0.008)	
**Right dorsal cingulum bundle**	0.39, 0.82	HV	A	0.53 (0.019)	3.8
		All_ALC	A	0.51 (0.013)	
**Left dorsal cingulum bundle**	2.06, 0.10	HV	A	0.61 (0.017)	4.9
		All_ALC	A	0.58 (0.012)	

^a^ The whole model is corrected for group, sex, age, and group by sex. ^b^ Groups not sharing the same letter are significantly different. ^c^ Standard error. ^+^ The observed significance of the whole model GLM in the splenium was the result of an effect of age, not group, on FA.

**Table 3:** Statistics of three-group Region of Interest analysis: HV vs ALC vs ALC+PTSD.

**Region of Interest**	**Whole Model** ^a^ **F ratio, Prob > F**	**Student’s t**			**% Difference in FA**		
		**Group**	**Level** ^b^	**Least Square Mean** (**SE** ^c^) **of FA**	**HV > ALC**	**HV > ALC+PTSD**	**ALC > ALC+PTSD**
**Anterior Commissure**	2.93, 0.017	HV	A	0.39 (0.020)	25.6	18.0	-10.3
		ALC	B	0.29 (0.022)			
		ALC+PTSD	B	0.32 (0.020)			
**Fornix**	4.90, 0.0006	HV	A	0.74 (0.019)	10.8	18.8	8.9
		ALC	B	0.66 (0.019)			
		ALC+PTSD	B	0.601 (0.019)			
**Genu**	2.84, 0.020	HV	A	0.84 (0.011)	2.4	3.6	1.2
		ALC	A	0.82 (0.011)			
		ALC+PTSD	A	0.81 (0.013)			
**Splenium^+^**	3.89, 0.0031	HV	A	0.86 (0.013)	0	1.2	1.2
		ALC	A	0.86 (0.015)			
		ALC+PTSD	A	0.85 (0.014)			
**Right IFO**	9.01, <0.0001	HV	A	0.62 (0.011)	11.3	8.1	-3.6
		ALC	B	0.55 (0.011)			
		ALC+PTSD	B	0.57 (0.011)			
**Right dorsal cingulum bundle**	0.50, 0.81	HV	A	0.53 (0.019)	7.5	1.9	-6.1
		ALC	A	0.49 (0.019)			
		ALC+PTSD	A	0.52 (0.019)			
**Left dorsal cingulum bundle**	1.37, 0.25	HV	A	0.61 (0.017)	4.9	6.6	1.7
		ALC	A	0.58 (0.019)			
		ALC+PTSD	A	0.57 (0.018)			

^a^ The whole model is corrected for group, sex, age, and group by sex. ^b^ Groups not sharing the same letter are significantly different. ^c^ Standard error ^+^ The observed significance of the whole model GLM in the splenium was the result of an effect of age, not group, on FA.

#### Anterior Commissure

HV had significantly higher FA than All_ALC (F = 11.04, p = 0.0017). The three-group analysis revealed that HV had significantly higher FA than ALC and ALC+PTSD (F = 5.88, p = 0.0053), but ALC and ALC+PTSD were not significantly different.

#### Fornix

HV had significantly higher FA than All_ALC (F = 18.88, p < 0.0001). The three-group analysis revealed that HV had significantly higher FA than ALC and ALC+PTSD (F = 11.28, p = 0.0001), but ALC and ALC+PTSD were not significantly different. In addition to group differences in the three-group analysis, females, regardless of group, had significantly higher FA than males (F = 5.25, p = 0.027) and older subjects, regardless of group, had greater FA than younger subjects (F = 4.12, p = 0.048).

#### Genu

Though alcohol dependent subjects had lower FA than HV subjects, these differences did not reach significance in either the two-group analysis (F = 3.0, p = 0.09) or three-group analysis (F = 1.82, p = 0.17). 

#### Splenium

No significant group differences in FA were found in the two-group analysis (F = 0.18, p = 0.67), but age was found to be a main effect (F = 13.54, p = 0.0006) and was negatively associated with FA, whereby younger subjects, regardless of group, had significantly higher FA. Similarly in the three-group analysis, no significant group differences in FA were found (F = 0.43, p = 0.65), but younger subjects had significantly higher FA than older subjects (F = 10.73, p = 0.0020). 

#### Right IFO

HV had significantly higher FA than All_ALC (F = 19.48, p < 0.0001), and younger subjects, regardless of group, had significantly higher FA than older subjects in the two-group analysis (F = 4.63, p = 0.037). The three-group analysis revealed that HV had significantly higher FA than ALC and ALC+PTSD (F = 11.34, p < 0.0001), with no significant differences between ALC and ALC+PTSD. In addition to group differences in the three-group analysis, younger subjects, regardless of group, had significantly higher FA than older subjects (F = 6.15, p = 0.017).

#### Left Dorsal Cingulum Bundle

Though alcohol dependent subjects had lower FA than HV subjects, these differences did not reach significance in either the two-group analysis (F = 2.48, p = 0.12) or three-group analysis (F = 1.27, p = 0.29). 

#### Right Dorsal Cingulum Bundle

No significant group differences were found in the two-group analysis (F = 1.17, p = 0.29) or three-group analysis (F = 1.21, p = 0.31). 

## Discussion

The present study applied a whole brain DTI analysis to examine the microstructure of major white matter pathways in healthy volunteers and alcohol dependent subjects with and without comorbid PTSD. TBSS results revealed widespread group differences in FA, primarily in commissural and limbic structures. We performed ROI analyses on tracts that showed significant group differences using the TBSS method, correcting for sex, age, and a group by sex interaction. In line with our hypothesis, healthy volunteers had significantly higher FA than alcohol dependent subjects in several white matter structures. Contrary to our hypothesis, there were no significant differences in FA between alcohol dependent subjects with and without PTSD in the TBSS analysis or the post-hoc ROI analysis. 

### Group Effects

The fornix was found to be the most significantly different region between healthy volunteers and alcohol dependent subjects. This midline white matter structure is involved in learning and memory formation, and connects the hippocampus to the mammillary bodies [43–46]. Previous studies have shown this major limbic pathway to have lower FA in alcoholism [20,24], linking it to the compromised motivational circuitry and deficits in learning and memory observed in alcoholics. Indeed, abnormal diffusivity measures in the fornix have been associated with poorer performance in cognitive and motor tasks [20]. Further, lower FA in the fornix and other projection and limbic fibers in adolescent alcohol and marijuana users predicted future substance use [47], arguing that reduced anisotropy in these structures may lead to increased risk-taking in adolescence [48].

We expected our ROI analysis of the bilateral dorsal cingulum bundle to show differences between control subjects and alcohol dependent subjects. Decreased white matter integrity of the cingulum bundle has been reported in alcohol dependence [19,20,24], as the cingulum plays an important role in learning and memory, emotional processing, and reward circuitry [49–51]. We also hypothesized that differences between ALC and ALC+PTSD would manifest in this white matter structure, as the anterior cingulate cortex (ACC) and cingulum have been implicated in PTSD previously. The anterior cingulate cortex (ACC), a grey matter structure with extensive connections with the amygdala, is found to be most adversely affected in PTSD; studies suggest that abnormal functioning of the ACC results in insufficient modulation of the characteristic hyperactive amygdala to traumatic stimuli in PTSD [33,52]. Kim et al. [33] were the first to examine whether the abnormalities seen in the ACC in PTSD could be attributed to abnormalities in the white matter pathway within this structure. In particular, they found decreased white matter integrity in the left cingulum bundle. Kim and colleagues [33,34] reasoned that this finding was in congruence with previous studies indirectly suggesting left hemisphere dysfunction in comparison to observed right hemisphere dominance in PTSD [53–55]. Accordingly, given the more right-sided deficits observed in alcoholism [5,13,19], these two competing right-sided effects in a combined ALC+PTSD population may average out, resulting in no observable difference between ALC and ALC+PTSD. This may also be an explanation for why no differences were seen between control subjects and alcohol dependent subjects, at least in the right dorsal cingulum bundle. It was surprising that no significant differences were seen in the left dorsal cingulum bundle, as this bundle is subject to both the detrimental effects of alcohol dependence as well as the indirect left hemisphere dysfunction in PTSD. 

Another white matter structure that had significantly lower FA in the alcohol dependent subjects was the right inferior fronto-occipital fasciculus (IFO). This long-range association fiber runs from the occipital lobe through the temporal lobe to the inferior and superior frontal gyri [56The IFO is thought to mediate top-down visual processing and recall [56–58]. Indeed, this tract has been shown to participate in some forms of memory, as higher FA in the right IFO within alcohol-dependent subjects was correlated with better visuospatial memory [24]. Yeh et al. [24] also performed a combination of seed analysis and probabilistic tractography to show that areas of lower FA in alcohol-dependent subjects were connected with major centers of the reward system through the IFO. Specifically, the IFO is connected with the inferior frontal cortex, an area responsible for executive function, and it also is a major tract within the amygdalo-hippocampal complex, a region important for learning and memory [24,59]. The IFO has been an area of interest in several adolescent studies of substance use as well. Jacobus et al. [60] found the IFO to have significantly lower FA in binge-drinking teens compared to healthy control teens. Interestingly in the present study, the right IFO was the only region that showed a significant difference between HV and ALC at the highest threshold of p = 0.003 in the TBSS analysis. Our results taken with those from previous findings suggest that the IFO may be a potential biomarker present from early problem drinking to later adult alcohol dependence. A longitudinal study is needed to further investigate these findings and determine if the IFO in fact plays a causal role in alcohol misuse.

To our knowledge, this is the first report showing a reduction in anisotropy of the anterior commissure (AC) in alcohol dependence and PTSD. The AC, an interhemispheric fiber pathway connecting the amygdalae of the temporal lobes and orbitofrontal cortices, is involved in a multitude of functions, including reward, decision-making, and cognitive processes [61]. We found this commissural tract to have lower FA in the alcohol dependent groups, suggesting changes in white matter microstructure. Previous studies have shown AC fiber degradation in thiamine deficiency [62], a condition commonly associated with alcohol dependence [63]. One study found that inhalant exposure in adolescent rats resulted in white matter abnormalities and delayed maturation in the AC, leading the authors to suggest that such a defect may contribute to suboptimal processing speed between the hemispheres [61]. A study on adolescent bipolar disorder (BPD) also found that decreased white matter anisotropy of the AC in BPD was associated with increased aggression, suggesting that the AC plays an important role in emotional processing [64]. 

The commissural fiber that has received the most attention in alcohol research is the corpus callosum (CC), the largest white matter pathway connecting the two cerebral hemispheres [65]. Studies investigating the CC in alcohol dependent populations have generally reported lower FA throughout the length of the commissure, including the genu and the splenium [11,18,21,25,66]. In contrast to these studies, we found no significant differences in FA between controls and alcohol dependent subjects in either structure. The lack of significance may be attributed to the age difference between out groups, as discussed below. 

### Age Effects

Age was found to be a main effect in the splenium, IFO, and fornix, whereby increasing age was correlated with lower FA in the splenium and IFO, but higher FA in the fornix. The results in the splenium and IFO are consistent with Lebel et al. [67] who examined the microstructure of major white matter pathways over the human lifespan and found FA to decrease with advancing age. Regarding the splenium, observing a lack of significant group differences in FA appears to have been due to the significant difference in mean age between groups. Whereas previous studies have had better age-matched controls, the mean age of our healthy volunteers was 26.2, compared with 34.8 of our combined alcohol dependent group (F = 18.12, p < 0.0001). Given that the splenium and genu have been found to reach peak FA at 24.8 and 20.8 years of age, respectively [67], and that in our study age was reaching trend level in the genu (two-group analysis: F = 2.67, p = 0.11) and was the only main effect in the splenium, supports the possibility that no group differences were observed because of the more significant effect of age. Regarding the fornix, our finding of increased FA with increasing age was rather surprising, since the fornix reaches its peak FA at the earliest age and declines slowly in integrity thereafter [67]. A closer look at the relationship between age and FA in the fornix revealed that this positive association was driven by the ALC+PTSD group, such that older ALC+PTSD subjects had higher FA than younger ALC+PTSD subjects. Despite the positive association, mean FA in the ALC+PTSD was significantly less than HV mean FA. 

### Sex Effects

Sex was found to be a main effect in the fornix in the three-group comparison. While we found significant sex effects in these two white matter regions, we did not observe a group by sex interaction in any of the regions examined. Controversy remains about whether alcohol’s damaging effects on white matter pathways are more pronounced in women than in men, but our results, along with more recent studies [11,20,25] do not lend support to that claim. 

### ALC versus ALC+PTSD

After finding no significant differences between ALC and ALC+PTSD in any structure, we performed post-hoc exploratory analyses between ALC and ALC+PTSD to look more closely at various factors influencing FA in these groups. Since recovery of FA has been observed with abstinence [66], we sought to investigate whether the significant difference between ALC and ALC+PTSD in their number of days abstinent influenced group FA. In a correlational analysis, we found no significant relationship between the two measures in any structure except the splenium, where, surprisingly, a longer duration of abstinence was correlated with lower FA values (R^2^ = 0.14, P = 0.037). This is in contrast to previous studies showing an increase in FA with abstinence from alcohol [66]. These previous studies, however, included subjects with much longer periods of abstinence (i.e. one year). Thus, we postulate that the reason we did not find the same results is because the subjects in our study abstained for a much shorter time period.

In addition to examining the potential effects of abstinence on FA, we also looked more closely at the relationship between PTSD severity and FA. A commonly used metric for PTSD severity is the Global Severity score from the Clinician-Administered PTSD Scale (CAPS) [68]. We specifically used subjects’ Global Severity score obtained at the start of their admission to our Inpatient Unit, thus reflecting PTSD severity before the onset of treatment. In a correlation analysis between FA and the Global Severity score of CAPS, no significant relationship was found in any ROI except the genu (F = 5.36, p = 0.041), whereby a higher CAPS score was associated with lower FA. Given that a mean CAPS score of 2.77 is a moderate-to-high PTSD severity score, we do not expect that weak severity of PTSD to be the reason for the absence in difference between the two alcoholic groups. However, assessing contribution of the finer CAPS scores with a larger PTSD group in future studies may reveal more specific differences between PTSD symptom severity and FA. 

The results of our study must be interpreted with caution, as there are some limitations that need to be considered. One limitation is that our subject groups were not exactly age- and sex-matched. Although we used age and sex as covariates to remedy this limitation, both were main effects in two structures, potentially masking group differences. With regard to finding no group by sex interactions in any structure, we cannot conclusively claim this finding since our groups did not have equal numbers of males and females. Therefore, even though power was relatively high when sex was found to be a main effect and approaching alpha level when it was not, we would caution the readers of over interpreting the sex differences. Additionally, alcoholics usually suffer from multiple psychotic and substance abuse comorbidities [69,70]. Given the sample size in this study, it was not possible to perform thorough statistical analyses to separate the effects, if any, of these comorbidities. A large number of alcoholics have also experienced elevated levels of stress during childhood [71,72]. It is known that early-life stress can have a profound impact on brain development by way of increased activation of stress hormones [73,74]. One study has shown that excess levels of the stress hormone glucocorticoid disrupt proper glial cell division, resulting in reduced myelination [75]. Our results lend support to these findings, since available data shows that all but one ALC+PTSD individual experienced childhood abuse, and mean measures on the Early Life Stress Questionnaire (ELSQ; scale from 0-19) were 4.2 for the combined alcohol dependent group compared to 0.4 for the healthy volunteer group (F = 27.4, p < 0.0001). When looking at the effects of ELSQ on FA, a significant relationship was found only in the anterior commissure, whereby a higher ELSQ score was surprisingly associated with greater FA. Incorporating ELSQ into the model with all covariates showed the same positive relationship between ELSQ and FA in the anterior commissure, but this relationship was no longer significant, as group was the only significant factor, suggesting that alcohol dependence contributes most to differences in FA. As stated above with PTSD severity, it is necessary to include a high ELSQ group with no alcohol dependence to better determine the separate effects of stress on FA. Lastly, the focus of this study was on effect of alcohol dependence and its comorbidity with PTSD on overall diffusion anisotropy. Examination of only FA cannot address the specific contributions from abnormal axonal and myelin microstructure to our results. A future study examining additional diffusivity measures in a larger sample size with better age- and sex-matched controls would give us a more complete understanding of alcohol’s effect on white matter integrity. 

## Conclusion

Our whole-brain DTI and post-hoc ROI analysis of healthy volunteers and alcohol dependent subjects with and without PTSD revealed widespread group differences in FA. In line with our hypothesis, we found the alcohol dependent subjects to have lower FA in several white matter structures, though no differences in FA were observed between the alcohol dependent subjects with and without PTSD. Specifically, the alcohol dependent subjects had lower FA in the fornix, anterior commissure, and right inferior fronto-occipital fasciculus, areas known to participate in emotional processing, cognition, and executive functioning. Our results suggest that some of the phenotypic and behavioral observations seen in alcoholics could be explained by microstructural abnormalities in these frontal and limbic structures.

## Supporting Information

Table S1 **Current Axis I Diagnoses for alcohol dependent subjects without PTSD (ALC) and alcohol dependent subjects with PTSD (ALC+PTSD).**
(DOC)
