# Supplementary material for: White Matter Microstructure Alterations: A Study of Alcoholics with and without Post-Traumatic Stress Disorder
Source: PLoS One. 2013 Nov 18;8(11):e80952. doi: 10.1371/journal.pone.0080952 (PMC3832443; doi:10.1371/journal.pone.0080952)
Supplement: Table S1 — Current Axis I Diagnoses for alcohol dependent subjects without PTSD (ALC) and alcohol dependent subjects with PTSD (ALC+PTSD). (DOC) [file pone.0080952.s001.doc]

**Table S1.**

| Current Axis I Diagnoses | ALC (n=19) | ALC+PTSD (n=17) |
| --- | --- | --- |
| Alcohol Dependence | 19 | 17 |
| Cocaine Abuse | 1 | 0 |
| Cocaine Dependence | 0 | 2 |
| Cannabis Abuse | 1 | 0 |
| Cannabis Dependence | 0 | 1 |
| Amphetamine Abuse | 0 | 0 |
| Amphetamine Dependence | 0 | 0 |
| Opioid Abuse | 0 | 0 |
| Opioid Dependence | 0 | 0 |
| Hallucinogen Abuse | 0 | 0 |
| Hallucinogen Dependence | 0 | 0 |
| Other Substance Abuse | 0 | 1 |
| Major Depressive Disorder | 2 | 3 |
| Other Mood Disorder (Dysthymia, substance-induced mood disorder, Bipolar) | 0 | 3 |
| Alcohol-Induced Mood Disorder | 3 | 4 |
| PTSD | 0 | 17 |
| Social Phobia | 3 | 3 |
| Generalized Anxiety Disorder | 3 | 0 |
| Alcohol-Induced Anxiety | 2 | 2 |
| Other/Unknown Substance-Induced Anxiety | 1 | 0 |
| Anxiety NOS | 1 | 0 |
| Other Anxiety | 1 | 0 |
| Specific Phobia | 1 | 0 |
| Eating Disorder | 0 | 1 |
| Obsessive-Compulsive Disorder | 0 | 1 |
